# A high-throughput expression screening platform to optimize the production of antimicrobial peptides

**DOI:** 10.1186/s12934-017-0637-5

**Published:** 2017-02-13

**Authors:** Christine Schreiber, Hagen Müller, Oliver Birrenbach, Moritz Klein, Doreen Heerd, Tobias Weidner, Denise Salzig, Peter Czermak

**Affiliations:** 1 University of Applied Sciences Mittelhessen, Wiesenstraße 14, 35390 Giessen, Germany; 2 Fraunhofer Institute for Molecular Biology and Applied Ecology (IME), Project group Bioresources, Giessen, Germany; 3 Faculty of Biology and Chemistry, Justus Liebig University Giessen, Giessen, Germany; 4 Department of Chemical Engineering, Kansas State University, Manhattan, USA

**Keywords:** Antimicrobial peptides, Expression screening, Recombinant protein production, Golden Gate cloning, Combinatorial library, *E. coli*, *P. pastoris*, Expression strain, Promoter, Fusion protein

## Abstract

**Background:**

Antimicrobial peptides (AMPs) are promising candidates for the development of novel antibiotics, but it is difficult to produce sufficient quantities for preclinical and clinical studies due to their toxicity towards microbial expression hosts. To avoid laborious trial-and-error testing for the identification of suitable expression constructs, we have developed a small-scale expression screening platform based on a combinatorial plasmid library.

**Results:**

The combinatorial library is based on the Golden Gate cloning system. In each reaction, six donor plasmids (each containing one component: a promoter, fusion partner 1, fusion partner 2, protease cleavage site, gene of interest, or transcriptional terminator) were combined with one acceptor plasmid to yield the final expression construct. As a proof of concept, screening was carried out in *Escherichia coli* and *Pichia pastoris* to study the expression of three different model AMPs with challenging characteristics, such as host toxicity or multiple disulfide bonds. The corresponding genes were successfully cloned in 27 *E. coli* and 18 *P. pastoris* expression plasmids, each in a one-step Golden Gate reaction. After transformation, small-scale expression screening in microtiter plates was followed by AMP quantification using a His_6_ tag-specific ELISA. Depending on the plasmid features and the expression host, the protein yields differed by more than an order of magnitude. This allowed the identification of high producers suitable for larger-scale protein expression.

**Conclusions:**

The optimization of recombinant protein production is best achieved from first principles by initially optimizing the genetic construct. The unrestricted combination of multiple plasmid features yields a comprehensive library of expression strains that can be screened for optimal productivity. The availability of such a platform could benefit all laboratories working in the field of recombinant protein expression.

**Electronic supplementary material:**

The online version of this article (doi:10.1186/s12934-017-0637-5) contains supplementary material, which is available to authorized users.

## Background

Antimicrobial peptides (AMPs) form part of the innate immune system in nearly all living organisms, and they act against a spectrum of pathogens including Gram-positive and Gram-negative bacteria, viruses and fungi. These small and often cationic peptides are therefore promising candidates for the development of alternative antibiotics [1]. The identification of a new AMP is often followed by chemical synthesis to produce sufficient amounts for initial small-scale functional tests. However, structural determination, toxicity testing and preclinical studies require larger amounts that cannot be produced in a cost-effective manner by chemical synthesis, and heterologous expression is therefore required. Heterologous expression is also necessary if the AMP is longer than ~50 amino acids or contains more than one disulfide bond [2]. Although recombinant protein expression is inexpensive and scalable, AMPs raise two important challenges: first, they are often toxic towards microbial expression hosts, and second, they often contain multiple disulfide bonds. One particular expression host is therefore unable to fulfill all conditions necessary to produce all classes of AMPs.

The bacterium *Escherichia coli* is widely used as an expression host because it is genetically well characterized, easy to manipulate, cultivation is simple and inexpensive, and there are several decades of experience in cloning, process scale-up and automation [3]. However, AMPs are often toxic towards *E. coli*. For example, glycine and proline rich coleoptericins, such as BR021 and BR023 from the harlequin ladybird *Harmonia axyridis*, have minimal inhibitory concentrations (MICs) of 63 and 125 µM, respectively [4, 5]. Similarly, gibberellin stimulated-like 1 (GSL1) and the deaminated derivatives of other AMPs such as MSI-344 are also toxic towards *E. coli* [6]. Toxic peptides can be produced in *E. coli* by genetic masking using a fusion protein, e.g. thioredoxin fusions disrupt the bactericidal activity of GSL1 [7]. Toxic peptides can also be expressed as inclusion bodies, as shown by the fusion of MSI-334 to PurF [6]. A third possibility is the use of specialized *E. coli* strains such as OverExpress C41 (DE3) and OverExpress C43 (DE3), which carry mutations making them more tolerant towards toxic proteins. SpStrongylocin 1 and 2 could not be expressed in *E. coli* BL21 (DE3), but were successfully produced in OverExpress C43 (DE3) [2]. The drawback of these strains is that their growth and production rates are often lower than in their non-engineered counterparts.

The expression of proteins with multiple disulfide bonds in *E. coli* is also challenging. A suitable model is the *Galleria mellonella* insect metalloproteinase inhibitor (IMPI), which is 69 amino acids in length and contains five disulfide bonds [8]. Two strategies are available for the production of such proteins in *E. coli*. The first uses genetically engineered strains with a non-reducing cytoplasmic milieu, achieved by mutations in the thioredoxin reductase (*trxB*) and glutathione reductase (*gor*) genes. Such strains include Origami B, Origami 2 and the Rosetta-gami B strains from Novagen. They tend to grow slowly, but they can successfully express disulfide-rich peptides such as Divercin V41 [9]. The second strategy involves targeting proteins to the periplasmic space. This can be accomplished by adding a signal sequence (e.g. from the MalE protein) but the success of this approach has been variable, as shown by the expression of disulfide-rich venom peptides with 2–6 disulfide bonds and lengths ranging from 17 and 67 amino acids [10].

An alternative expression platform that forms disulfide bonds naturally is the yeast *Pichia pastoris* [11]. This species is advantageous compared to model yeasts such as *Saccharomyces cerevisiae* because it benefits from powerful inducible promoters and is suitable for high-density cultivation. Furthermore, most peptides that are toxic towards Gram-negative bacteria such as *E. coli* are not toxic in *P. pastoris* and vice versa. AMPs that have been produced successfully in *P. pastoris* include snaking-1, a 63-amino-acid cysteine-rich plant AMP with six disulfide bonds [12]; clavanin, a 23-amino-acid α-helical AMP that is toxic towards both Gram-positive and Gram-negative bacteria [13]; and cecropin D, a 3.9-kDa AMP that is post-translationally modified [14]. AMP toxicity in *P. pastoris* can also be masked with fusion proteins, so including such genetic elements in an expression plasmid can have a significant impact on AMP yields. Such elements may not only alter AMP expression but also facilitate protein detection and purification, which is beneficial because downstream processing represents up to 80% of recombinant protein production costs. If fusion proteins are used, protease cleavage sites for the removal of fusion tags must also be included [15].

The optimal genetic setup for each target peptide and host is necessary to achieve high yields, and this requires the most appropriate combination of promoter, fusion protein partner and protease cleavage site. However, the rational prediction of optimal combinations is not usually possible. Expression screening is therefore necessary, and must address the following requirements: (1) screening must be rapid; (2) the procedure must be standardized and independent of the DNA sequence so that the same protocol can be used for every new target protein without further modifications; and (3) screening should be adaptable to different expression hosts, such as *E. coli* and *P. pastoris*.

**Fig. 1:**
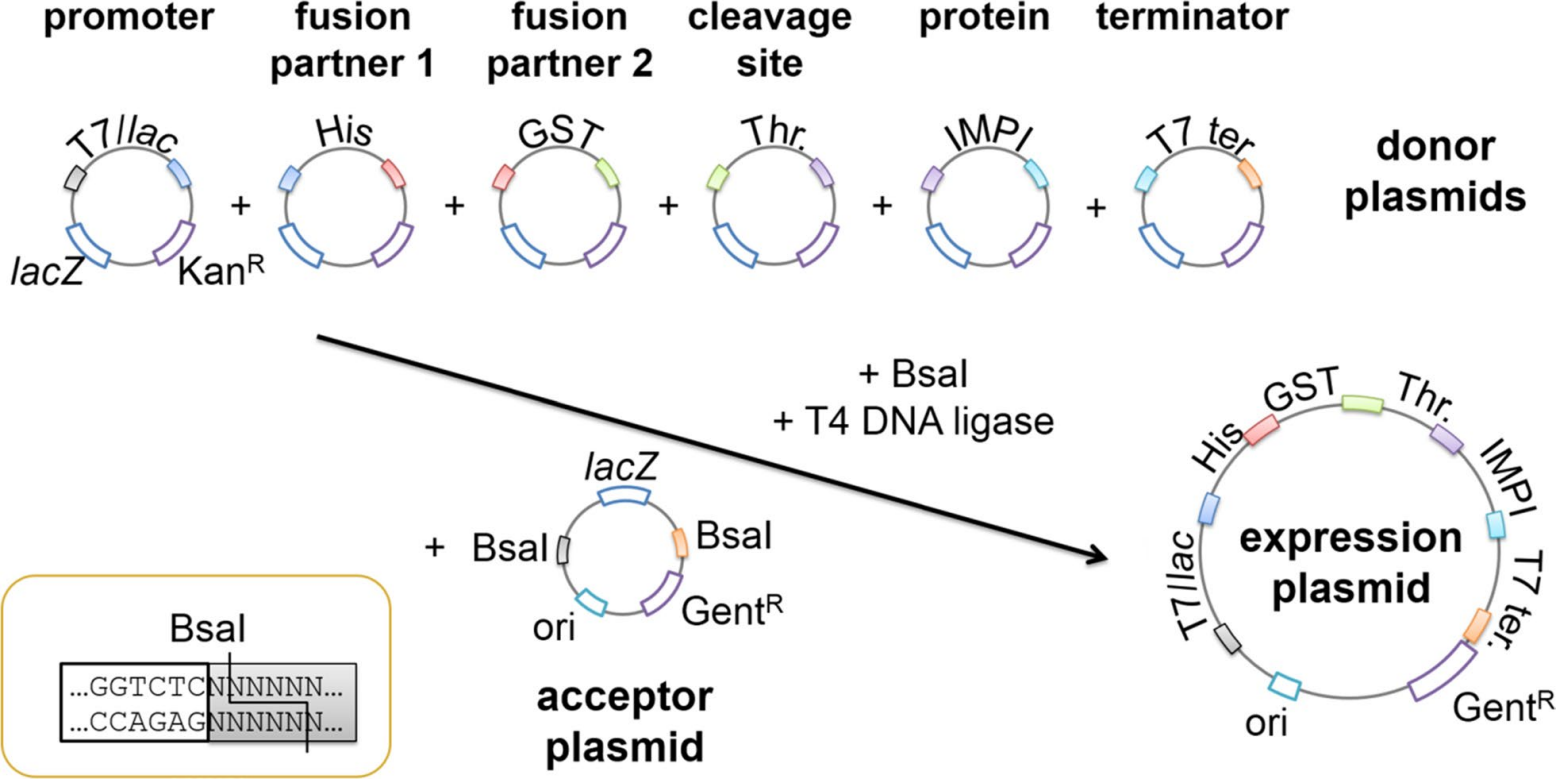
Golden Gate assembly of the expression plasmid in a single reaction. Six donor plasmids and one acceptor plasmid were combined to form each expression plasmid by directional cloning in a restriction-ligation step using the endonuclease *Bsa*I and T4 DNA ligase. The overhangs produced by *Bsa*I are colored

Several small-scale expression screening platforms have been reported recently [16, 17]. Most comprise a pool of pre-assembled (often commercially available) expression plasmids that carry different promoters, fusion partners, affinity tags and cleavage sites. The gene of interest is usually introduced into the plasmids by restriction-ligation at a multiple cloning site or by homologous recombination. A widely-used example is the Gateway® system (Thermo Fisher Scientific) although one disadvantage is the limited combination of features from a small pool of commercially available plasmids, which reduces the number of combinations that can be screened. However, for a screening system highly flexible expression vectors are required which led to the development of combinatorial cloning techniques featuring modular expression plasmids [3]. Methods that allow the simultaneous assembly of multiple expression plasmids from diverse components include restriction-free cloning [18], sequence and ligation independent cloning (SLIC) [19, 20], and Golden Gate cloning [21, 22]. Restriction-free cloning and SLIC are based on the polymerase chain reaction (PCR). Therefore, custom primers must be designed for every new protein of interest and additional cloning steps are necessary that are prone to mutations. In contrast, Golden Gate cloning does not require preparative PCR steps and allows the one-step assembly of multiple DNA sequences in a given order and orientation [21]. Golden Gate cloning makes use of type IIs restriction enzymes such as *Bsa*I that cut at sites adjacent to their recognition sequences, allowing the selection of any four-nucleotide 5′ overhang for ligation and thus the restriction and ligation of multiple DNA sequences in a single cloning step (Fig. 1).

Here we describe a straightforward screening platform based on the creation of tailor-made expression plasmids by Golden Gate cloning without combinatorial limitations [23, 24]. We defined six classes of plasmid features that are combined into expression plasmids in a single step (Fig. 1): the promoter system, two possible fusion partners, the protease cleavage site, the target protein and the transcriptional terminator. Each expression plasmid feature is modular and can be assembled independently with every other feature in the corresponding library leaving only a four-nucleotide “scar” between elements (or two amino acids in the coding region). The modular platform maximizes flexibility for the combination of conventional and also newly discovered features. It will be particularly useful for smaller laboratories lacking complex technical equipment such as pipetting robots, but is also suitable for industrial expression screening approaches. The platform is universal because it can be applied to a broad range of protein types and expression hosts. To showcase the platform, we investigated the expression of three model recombinant AMPs with different characteristics: (1) IMPI (7.7 kDa; with five disulfide bonds) [8]; (2) BR021 (8.4 kDa; toxic towards *E. coli*) [4]; and (3) an antifungal peptide (AFP) from *Lucilia sericata* (8.2 kDa; without disulfide bonds and non-toxic to our hosts) [25]. We used different promoters and fusion proteins to demonstrate their impact on the protein yield. Expression screening was carried out in different *E. coli* and *P. pastoris* strains.

## Results and discussion

### Golden Gate assembly of a basic expression plasmid library in *E. coli*

Figure 1 shows the concept of the Golden Gate plasmid library. Six donor plasmids and one acceptor were combined to generate a panel of expression plasmids in a single restriction-ligation reaction. The *E. coli* cloning strain NEB10β was then transformed and blue-white screening was carried out to identify white colonies, which had incorporated an expression plasmid. It was necessary to optimize the Golden Gate reaction conditions to generate the modular plasmid library so the cloning efficiency was therefore compared using *Bsa*I-HF and the wild-type restriction enzyme *Bsa*I.

The proportion of white colonies after transformation was considerably higher when we used the wild-type enzyme *Bsa*I (97%) rather than *Bsa*I-HF (31%). The better performance of the wild-type enzyme may be caused by its full activity at 50 °C during the final restriction step, in contrast to the depleted activity of *Bsa*I-HF (NEB, personal communication). This step is necessary for the efficient digestion of residual acceptor plasmids because any remaining acceptor plasmids in the mixture produce blue colonies.

**Fig. 2:**
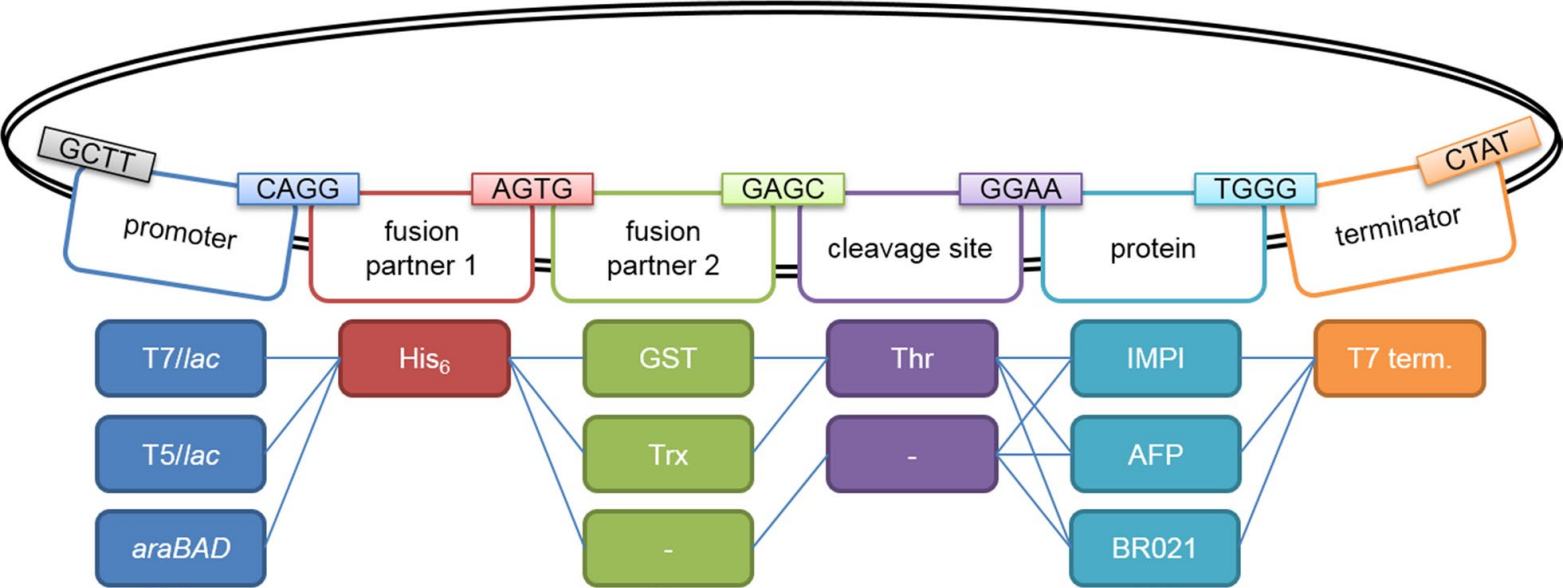
Basic plasmid library for *E. coli*. The plasmid library consists of 13 donor plasmids, resulting in 27 different expression plasmids. Three promoters (T7/*lac*, T5/*lac* and *araBAD*) were combined with a His_6_ tag and either GST or Trx as a fusion partner (or no fusion partner). A thrombin cleavage site was also included in the constructs with a fusion partner. The library includes three AMP genes encoding IMPI, BR021 or the *L. sericata* antifungal peptide (AFP). The T7 transcriptional terminator was present in all constructs. The chosen *Bsa*I overhangs are shown above the elements

Using the established Golden Gate assembly process, a plasmid library was constructed to express the three peptides of interest: IMPI, BR021 and AFP (Fig. 2). Three different promoters (T7/*lac*, T5/*lac* and *araBAD*) were combined with glutathione-*S*-transferase (GST) or thioredoxin (Trx) as fusion partners (or with no fusion partner). The three promoters were chosen because of their different strengths. The T7/*lac* promoter is a strong promoter and may therefore lead to the highest total protein yields. On the other hand, a strong promoter like T7/*lac* is associated with a higher risk of inclusion body formation [26] and is therefore not always the best choice when soluble expression is desired. All constructs contained an N-terminal His_6_ tag to facilitate detection and purification. The plasmids incorporating fusion partners also contained an additional thrombin cleavage site. We chose thrombin for cleavage because it is cheap and therefore suitable for large scale purification. The T7 transcriptional terminator was used in all plasmids. This generated a potential total of 27 expression plasmids, 18 of which contained all six elements.

**Fig. 3:**
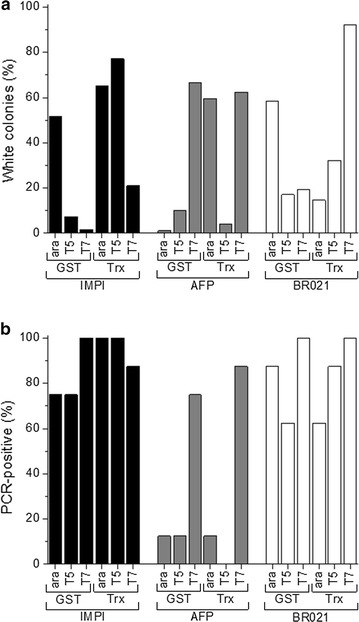
Proportion of white colonies and PCR-positive white colonies after the Golden Gate assembly of different *E. coli* expression plasmids. **a** The proportion of white colonies was determined in a blue-white screening assay. The proportion of white colonies varied according to which plasmid features were used. For each assembly, eight white colonies were analyzed by colony PCR. **b** The proportion of analyzed colonies with a PCR product of the correct size

For 17 of the 18 constructs ≥1 clone with correct band pattern was identified in a colony PCR analysis based on eight white colonies. The remaining plasmid (T5 promoter, thioredoxin and AFP) was detected after a second PCR run testing seven more colonies, which resulted in two hits. For all assemblies, we determined the proportion of white colonies and the proportion of colonies testing positive by colony PCR (Fig. 3a, b). The overall cloning efficiency was then calculated as the product of the proportion of white colonies multiplied by the proportion of colonies testing positive by colony PCR (Eq. 1):

1 $$ overall\;cloning\;efficiency = \frac{white\;colonies}{all\;colonies}*\frac{positive\;in\;colony\;PCR}{number\;of\;colony\;PCR\;samples} $$

**Fig. 4:**
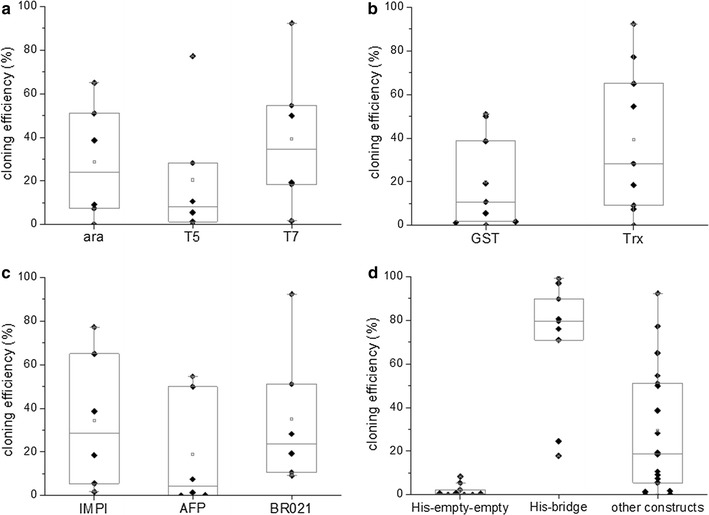
Cloning efficiencies for the Golden Gate assembly of different *E. coli* expression plasmids. Each dot represents one cloning procedure with an individual expression plasmid. There were no significant differences in the cloning efficiencies of the different promoters (**a**), fusion partners (**b**) or proteins (**c**). **d** Shows the cloning efficiencies for an expression plasmid where two consecutive plasmid features were left out, here the fusion partner and the protease cleavage site. In this case, Golden Gate cloning using two short dummy fragments was compared to a workaround strategy in which one of the neighboring plasmid feature sequences (here for the His_6_ tag) carries a *Bsa*I overhang directly complementary to the plasmid feature “protein of interest” so that no short dummy fragments are needed. As a comparison, the cloning efficiencies for the assembly of the other 18 expression plasmids, which carry all six classes of plasmid features, are also shown

Sequencing confirmed the positive events identified by colony PCR, so no further calculations were needed to describe the overall cloning efficiency. There was also no significant deviation in the cloning efficiency when using different promoters, fusion partners and target proteins (Fig. 4a–c). The Golden Gate assembly therefore appears to be a robust method regardless of the sequences of the individual plasmid features.

However, the cloning efficiencies achieved above could not be reproduced during the assembly of the nine expression plasmids lacking the fusion partner and protease cleavage site. To fill the gaps, we initially used two donor plasmids containing short dummy sequences flanked by the appropriate *Bsa*I recognition sites and overhangs. The cloning efficiencies of the expression plasmids containing two consecutive dummy fragments were poor (Fig. 4d; His-empty-empty). Therefore, three of the nine plasmids could not be assembled using the standard procedure, including the analysis of eight white colonies by colony PCR. This phenomenon may reflect the length of the dummy fragments. Because the fragments consist solely of the flanking overhangs and two additional base pairs, they are present as single strands under our reaction conditions (16 °C for ligation and 37 °C for restriction). Equation 2 can be used to calculate the melting temperature (T_M_) of short DNA molecules in solution, and this suggests that the dummy fragments have a T_M_ of 12 °C.

2 $$ {\text{T}}_{\text{M}} = \, \left( {{\text{A}} + {\text{T}}} \right) \cdot 2\,^\circ {\text{C }} + \, \left( {{\text{G}} + {\text{C}}} \right) \cdot 4\,^\circ {\text{C }} + { 8}\,^\circ {\text{C}} $$

Despite the low cloning efficiency, positive clones were usually identified by increasing the number of colonies screened by PCR. Alternatively, when a special combination of fragments such as “His-empty-empty” is required more frequently, a workaround strategy can be used in which a combined fragment is introduced as an additional donor plasmid, e.g. His_6_ with *Bsa*I overhangs complementary to the promoter system on one side and to the target protein on the other side. This workaround significantly increased the cloning efficiency (Fig. 4d; His-bridge) and the three expression plasmids that could not be assembled using the dummy donor plasmids based on the screening of eight white colonies were cloned successfully using this approach.

### Golden Gate assembly of a basic expression plasmid library in *P. pastoris*

**Fig. 5:**
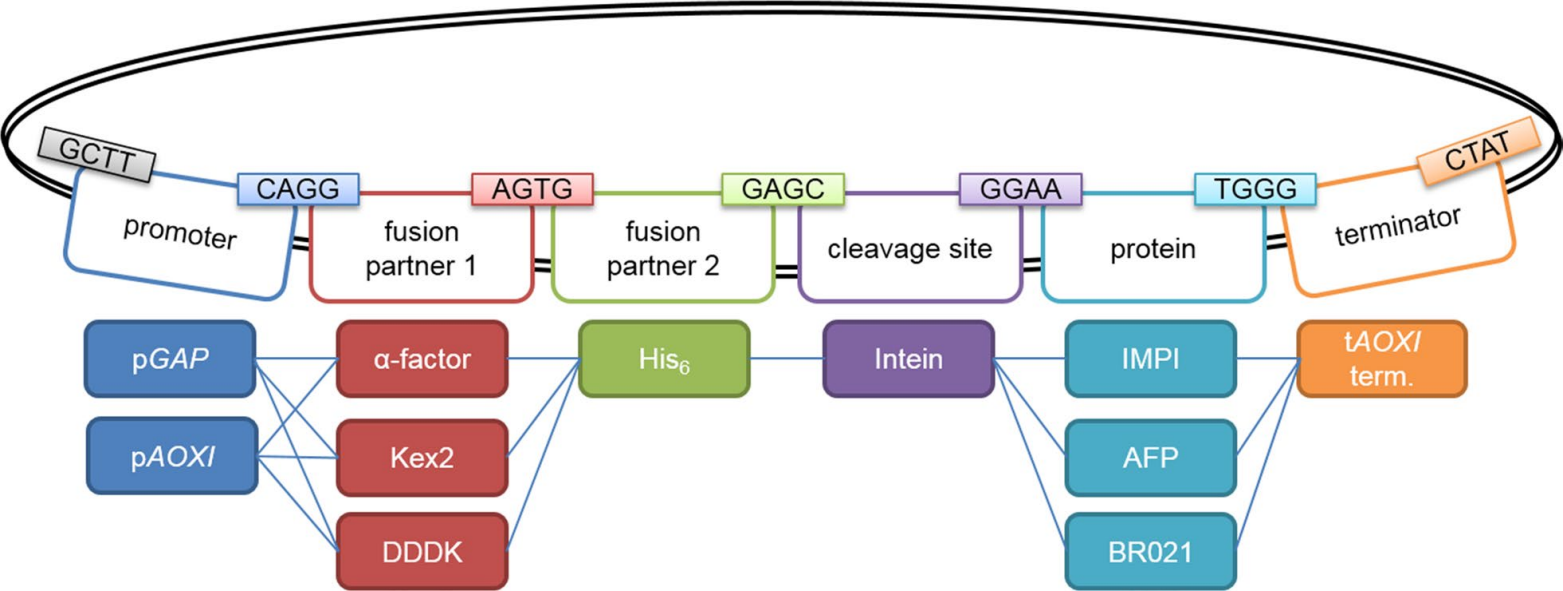
Basic plasmid library for *P. pastoris*. The plasmid library consists of 11 donor plasmids, resulting in 18 different expression plasmids. Two promoters (pGAP and pAOXI) were combined with three secretion factors (the α-mating factor, Kex2, DDDK), a His_6_ tag and an intein sequence. The tAOXI transcriptional terminator was present in all constructs

**Fig. 6:**
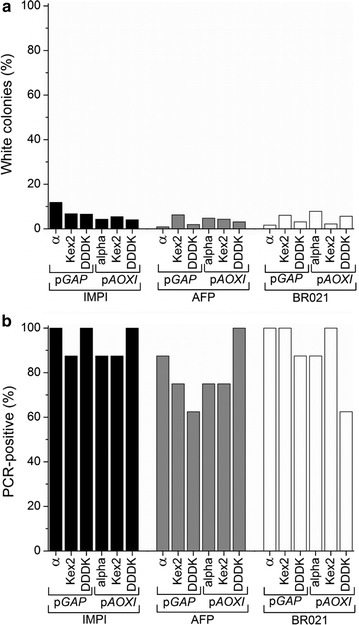
Portion of white colonies and PCR-positive white colonies after the Golden Gate assembly of different *P. pastoris* expression plasmids. **a** The proportion of white colonies was determined in a blue-white screening assay. The proportion of white colonies varied according to which plasmid features were used. For each assembly, eight white colonies were analyzed by colony PCR. **b** The proportion of analyzed colonies with a PCR product of the correct size

A second plasmid library was created in *P. pastoris* to achieve the expression and secretion of the peptides IMPI, AFP and BR021 (Fig. 5). This library combined two different promoters (the glyceraldehyde-3-phosphate dehydrogenase promoter p*GAP* and the alcohol oxidase I promoter p*AOXI*) with three different secretion factors (the α-mating factor, the same factor with an additional serine endoproteinase cleavage site (Kex2), and the secretion factor of a protein containing four tandem DDDK sequences). Each construct also contained a His_6_ tag to facilitate detection, a split intein for purification, and finally the transcriptional terminator from the alcohol oxidase I gene (t*AOXI*). All 18 expression plasmids were successfully generated by Golden Gate cloning followed by the transformation of *E. coli* and colony PCR analysis of eight white colonies. Figure 6 shows the cloning results in detail.

### Comparison of cloning efficiencies for *E. coli* and *P. pastoris*

The average percentage of white colonies was 37 ± 30% for the *E. coli* constructs and 5 ± 2.6% for the *P.* *pastoris* constructs. The average percentage of colonies displaying the anticipated band pattern following colony PCR analysis was 69 ± 35% for the *E. coli* constructs and 87 ± 13% for the *P.* *pastoris* constructs. The cloning efficiencies we observed were therefore lower than those reported by Engler et al. [22] using a similar protocol and the same *Bsa*I overhangs (99% white colonies and 94% colonies with the correct restriction pattern). The difference in cloning efficiency is therefore likely to be caused by the genetic elements we used, and can potentially be attributed to the vast difference in size between the genetic elements (T5/*lac* promoter: 1311 bp, His_6_ tag element: 21 bp) compared to the genetic elements used by Engler et al. which had all nearly the same size [22]. However, further investigations on this topic have to be done. Nevertheless, all 45 expression plasmids were cloned and verified by sequencing within 2 weeks, which confirms that the method is rapid and robust enough for our purpose.

### Expression screening in *E. coli*

The 27 expression plasmids assembled by Golden Gate cloning were used to screen for the optimal expression of IMPI, BR021 and AFP. Six *E. coli* expression strains with different characteristics were used. Rosetta-gami 2 (DE3) pLysS, Origami 2 and SHuffle T7 Express lysY are strains with an oxidizing milieu in the cytoplasm and were therefore selected for the expression of IMPI with its five disulfide bonds. OverExpress C41 (DE3) pLysS and OverExpress C43 (DE3) pLysS are strains that are suitable for the expression of toxic proteins [27] and were therefore chosen for the expression of BR021. The standard expression strain BL21 (DE3) was also included. We developed a small-scale screening approach suitable for any laboratory without pipetting robots and automation, which involved the rational selection of the two or three most promising strains for every expression plasmid (78 combinations). For two of these combinations the transformations failed, resulting in 76 viable combinations. A possible explanation for the failure of the two transformations could be the instability of the plasmids in the corresponding strains. In this case the combinations will not be useful for protein expression and can be excluded from the screening. Screening was carried out in 96-well microtiter plates. The cultures were grown from OD_600_ = 0.1 for 3 h at 37 °C, followed by the induction of protein expression and incubation for 17 h at 30 °C. Protein yields in the soluble fraction after cell lysis were determined by a sandwich ELISA using the His_6_ tag to identify the high producers.

**Fig. 7:**
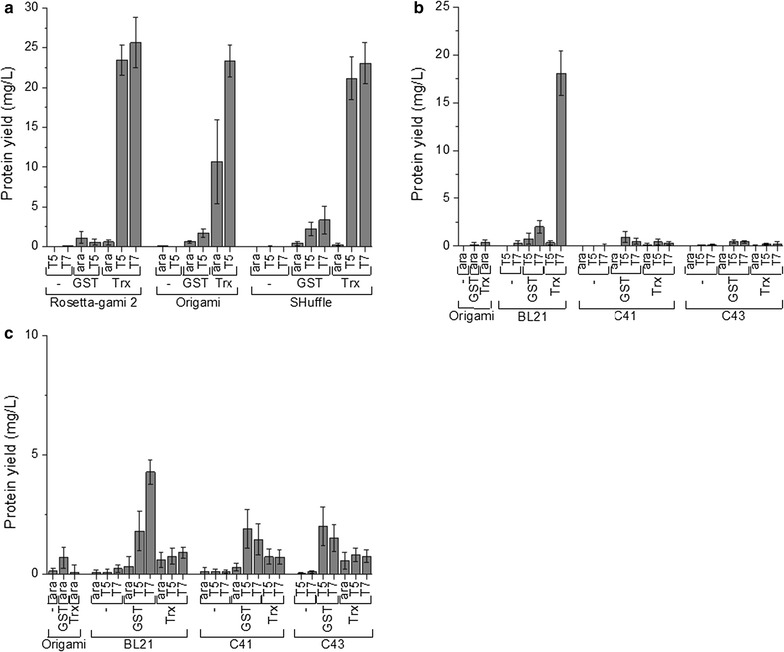
Expression screening in *E. coli*. Expression screening was carried out in 96-well microtiter plates to identify high producers for three different target proteins. The promoter, protein fusion partner and *E. coli* strain were varied. **a** IMPI, **b** BR021, **c** AFP. *Rosetta-gami 2* Rosetta-gami 2 (DE3) pLysS, *Origami* Origami 2, *SHuffle* SHuffle T7 Express lysY, *BL21* BL21 (DE3), *C41* OverExpress C41 (DE3) pLysS, *C43* OverExpress C43 (DE3) pLysS

As shown in Fig. 7, all IMPI high producers (yields of soluble IMPI > 10 mg/L_culture_) carried Trx as the fusion partner, whereas the yields were lower with GST as the fusion partner and lower still when the fusion tag was omitted. Similar results were reported previously for the comparison of Trx + His_6_, GST + His_6_ and the His_6_ tag alone [28]. The solubility-enhancing effect of the fusion partner was previously shown to depend on the size of the target protein and is most prominent for small proteins [29], which explains the substantial effect of fusion partners on the yield of the small (7.7 kDa) target protein IMPI. The promoter had a strong effect on expression as expected, and the inducible T7/*lac* promoter resulted in the greatest improvement in the yield of IMPI as previously noted in a study comparing the T7/*lac* and the *araBAD* promoters among others [30]. In contrast, we found that the expression strain had only a minor effect on the yield of IMPI.

For the toxic protein BR021, only one strain achieved a significant production level, highlighting the need for a screening strategy when dealing with difficult-to-express proteins. BR021 was successfully expressed in the standard expression strain BL21 as a Trx-fusion protein under the control of the T7/*lac* promoter system. Interestingly, only low yields were achieved in OverExpress C41 and C43 although these strains are specialized in the expression of toxic proteins in *E. coli*. To exclude the possibility of a false-positive result, BR021 expression using strain BL21 with the T7/*lac* + Trx combination was repeated in shaking flasks. Quantification of the soluble and insoluble protein fractions revealed that the majority of the target protein was present as insoluble inclusion bodies and only a small amount of soluble protein was present (Additional file 1). It is likely that the protein detected in the soluble fraction was only solubilized during cell lysis. Because the yield of insoluble protein was extremely high (~0.6 g/L_culture_) we will investigate the purification of Trx-BR021 from the inclusion bodies in future experiments.

The maximum yield of *L. sericata* AFP (4.2 ± 0.5 mg/L_culture_) was achieved in BL21 cells as a GST-fusion protein under the control of the T7/*lac* promoter. The successful expression of this AFP in *E. coli* BL21 (DE3) cells has previously been achieved using an N-terminal Trx-His_6_ tag [25].

### Expression screening in *P. pastoris*

*Pichia pastoris* was used as an alternative expression host in our screening platform because it is ideal for proteins that are too toxic or complex for production in *E. coli*. Expression was facilitated by including its two best-characterized promoter systems: the methanol-inducible p*AOXI* promoter, which is strictly regulated and allows the production of toxic proteins, and the constitutive p*GAP* promoter, which allows the application of simpler fermentation conditions [31].

*Pichia pastoris* secretes recombinant proteins into the fermentation broth if an appropriate secretion signal is included in the construct, thus facilitating protein purification. A protease cleavage site is also needed, to allow the recovery of the native protein without the signal sequence. We therefore combined the three AMPs with three different secretion factors (Fig. 5):

1. The α-mating factor [32]. This is one of the most widely used secretion factors, but in vivo processing can lead to the fragmentation of peptides that have internal accessible KR or RR dibasic amino acids.
2. The DDDK secretion factor [33]. This 18-amino-acid secretion factor was identified recently [33] and secrets a *P. pastoris* protein containing four DDDK tandem repeats. The α-mating factor and DDDK achieved similar secretion efficiency when tested with porcine carboxypeptidase and the *Erythrina caffra* trypsin inhibitor.
3. The α-mating factor with an additional Kex2 protease site [34]. Some proteins are secreted with low efficiency by the α-mating factor because the Kex2 site is inaccessible, and this can be addressed by adding a second site for the Kex2 protease.

To facilitate the removal of the fusion protein, we incorporated self-splicing inteins in the *P. pastoris* system rather than the classical thrombin cleavage site used in *E. coli*. Furthermore we investigated two different production strains: the X-33 wild-type strain and the SuperMan_5_ strain (*HIS* ^+^ *, pep4*Δ) in which selected peptidases are knocked out. After successful cloning using the Golden Gate system as described above, the two *P. pastoris* strains were transformed with the expression plasmids by electroporation. Whereas protein expression in *E. coli* is usually based on an episomally-maintained plasmid under constant selection, in *P. pastoris* the plasmid integrates into the genome and no selection is necessary during fermentation. Although integration is site-specific, the number of integrated copies is random and we therefore screened eight clones for each expression construct.

**Fig. 8:**
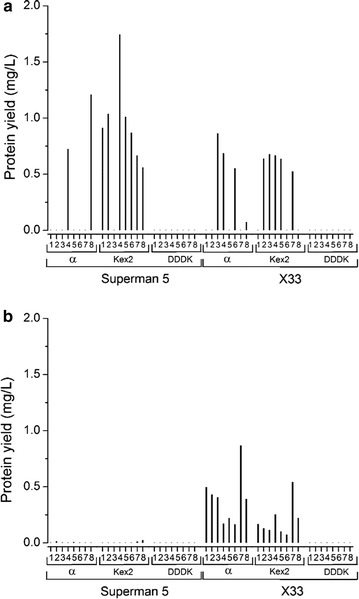
Expression screening for IMPI production in *P. pastoris*. Expression screening was carried out in 96-well microtiter plates to identify high producers. The secretion factor was varied in the screening and eight different clones for each combination were screened. **a** pGAP, **b** pAOXI

Expression in 96-deep-well plates was followed by His_6_ tag-specific ELISA to determine the AMP yields. We found that BR021 was not expressed at all, and only one clone was isolated expressing the *L. sericata* AFP but the yield was very low (0.17 mg/L_culture_). This clone comprised the constitutive pGAP promoter combined with the Kex2 secretion factor element in the SuperMan_5_ strain. The low yields of BR021 and AFP probably reflect the presence of proteases such as proteinase A (PrA) and proteinase B (PrB) [35], or they may not be properly secreted despite the presence of a secretion factor in the construct. In contrast, IMPI was expressed successfully by *P. pastoris* (Fig. 8). Only the DDDK secretion factor was incompatible with the expression of this peptide, whereas the α-mating factor achieved efficient secretion in the presence and absence of the additional Kex2 site. IMPI was successfully expressed in both *P. pastoris* strains under the control of the constitutive p*GAP* promoter, but only in strain X33 under the inducible p*AOXI* promoter. The highest yield was achieved with the p*GAP* promoter and Kex2 as the secretion signal in the SuperMan_5_ strain (1.7 mg/L_culture_).

### Comparison of expression screening in *E. coli* and *P. pastoris*

Expression screening in *E. coli* and *P. pastoris* achieved different outcomes for the three model AMPs. For BR021, expression as a soluble peptide was not possible, at least not with the genetic elements and strains we used. *E. coli* appears to be the most suitable expression host, and the purification of BR021 from inclusion bodies appears to be the most appropriate expression strategy. The *L. sericata* AFP could not be expressed at significant levels in either *E. coli* or *P. pastoris*, indicating that the screening platform must be expanded to include more genetic elements, additional strains or even further expression hosts. IMPI was expressed at high levels in both *E. coli* and *P. pastoris*, with yields depending on the strain and plasmid combination. We identified genetic elements compatible with high expression levels, e.g. the T5 and T7 promoter as well as Trx as fusion partner in *E. coli*, and the α-mating factor with or without the additional Kex2 site in *P. pastoris*. In a direct comparison, *E. coli* achieved higher IMPI yields than *P. pastoris* at the small screening scale using the combination of plasmid features we tested. However, large-scale and high-cell-density fed-batch or continuous fermentation tests would be necessary to determine which expression system is better for the respective AMP.

## Conclusions

Antibiotic-resistant bacteria are becoming more prevalent, and the demand for new classes of antimicrobial drugs is therefore increasing. Insect AMPs are promising candidates but the first challenging step is the heterologous expression of sufficient quantities of AMPs for preclinical and clinical studies. AMPs have diverse characteristics and expression screening is therefore necessary to identify the optimal combination of expression construct and host to achieve high yields. We developed a screening platform based on a combinatorial plasmid library, and screened for the optimal expression of three model AMPs in two hosts: *E. coli* and *P. pastoris*. Our results highlight the importance of screening before further process optimization, because different outcomes were observed for each AMP. IMPI was successfully expressed using different plasmid/strain combinations in both *E. coli* and *P. pastoris* and can now be produced at larger scales, whereas BR021 could only be expressed as insoluble inclusion bodies in *E. coli* and the *L. sericata* AFP could not be expressed using any of our strain/plasmid combinations. The latter case shows the limitations of small-scale screening with a limited number of combinations—it is possible that no hits will be achieved and subsequent larger screens may be necessary incorporating additional plasmids, strains and even host organisms. Our screening platform can be expanded to encompass additional hosts with little further effort, potentially including insect cells because they are more closely aligned with the native production of insect AMPs [36], and alternative yeasts such as *Kluyveromyces lactis* [37]. We are convinced that the platform will also be useful for larger proteins (>10 kDa), because Golden Gate cloning has been shown to work for DNA fragments up to at least 3500 bp [38] and the screening itself relies on simple cultivation in a 96-well plate followed by protein quantification using a His_6_-specific ELISA, which is a method that is commonly used for proteins of all sizes.

## Methods

### Cultivation of *P. pastoris* and *E. coli*

Unless stated otherwise, all culture media and ingredients for screening in *P. pastoris* were prepared according to the protocol from the Pichia Protein Expression Kit [39], although we replaced the BMGY medium with BMDY medium (containing glucose instead of glycerol).

*Escherichia coli* was cultivated in standard LB medium containing 10 g/L tryptone, 5 g/L yeast extract and 10 g/L NaCl at pH 7.0 (Carl Roth).

### Constructs

The donor plasmid was derived from the commercial plasmid pJ915 (DNA2.0) and contains an ILV5 promoter, an EM72 promoter, a bleocin-resistance cassette, the AOD terminator, the pUC origin of replication and a *lac*Z cassette. The plasmid features were synthesized by GenScript. GenScript used the PciI restriction site in plasmid pUC57-Kan as a cloning site to insert the plasmid features into the vector. The DNA sequences for the plasmid features were flanked by *Bsa*I recognition sites to allow directional cloning. Two extra base pairs were added upstream of the DNA sequences for the protein fusion partners, protease cleavage sites and AMP genes to avoid frameshifts. The four-nucleotide *Bsa*I overhang sequences were adopted from Engler et al. [22] (Fig. 2). The synthesized donor plasmids were used directly for Golden Gate cloning.

### Golden Gate cloning

Each Golden Gate cloning reaction required 100 ng of the acceptor plasmid (pJ915-lacZ), 100 ng of each donor plasmid, 2.5 U *Bsa*I/*Bsa*I-HF and 300 U T4 DNA ligase (NEB, 2000 U/µL) in a reaction mixture of 15 µL in 1× T4 DNA ligation buffer (NEB). The reactions comprised 50 temperature cycles (2 min at 37 °C for digestion, 5 min at 16 °C for ligation) followed by 10 min final restriction at 50 °C and 20 min heat inactivation at 65 °C.

After the Golden Gate reaction, chemically competent *E. coli* 10-beta (NEB) cells were transformed with the reaction mix using the following heat shock protocol. First 80 µL of cells were thawed on ice and 5 µL of reaction mix were added. Afterwards the mix was incubated on ice for 15 min. Then a heat shock at 42 °C for 1 min was done. Afterwards 250 µL LB medium were added and the cells were incubated at 600 rpm and 37 °C for 45 min. Transformants were plated on selective LB agar plates containing either 15 µg/mL gentamicin or 10 µg/ml Bleocin™, 1 mM IPTG and 100 µg/mL X-Gal for blue–white screening. After incubation for 1 day at 37 °C, the blue and white colonies were counted.

Eight colonies per construct were analyzed by colony PCR using primers that bind to the acceptor plasmid backbone. Colony PCR was carried out using OneTaq DNA Polymerase (NEB) following the manufacturer’s protocol. Plasmid DNA was isolated from colonies with a correct colony PCR band pattern using the NucleoSpin® Plasmid EasyPure kit (Macherey–Nagel). The DNA was sequenced by GATC biotech with the same primers used for colony PCR.

### Transformation of *E. coli* expression strains

Six different *E. coli* strains were used for expression screening: Rosetta-gami 2 (DE3) pLysS (Merck Millipore), Origami 2 (Merck Millipore), BL21 (DE3) (NEB), SHuffle T7 Express lysY (NEB), OverExpress C41 (DE3) pLysS (Sigma-Aldrich), OverExpress C43 (DE3) pLysS (Sigma-Aldrich). *E. coli* expression strains were transformed with the expression plasmids by heat shock. An 80-µL aliquot of competent cells was thawed on ice before adding 5 µL of the Golden Gate mixture. The cells and DNA were incubated for 15 min on ice and heat shocked for 1 min at 42 °C. After the heat shock, 250 µL LB medium was added to the transformants and the mixture was incubated for 45 min at 37 °C, shaking at 1100 rpm in a thermomixer (Eppendorf). After the incubation, 50 µL of the cell mixture was spread on LB agar containing 15 µg/mL gentamicin.

Prior to expression screening, cryocultures were prepared from overnight liquid cultures grown in LB containing 15 µg/mL gentamicin at 37 °C and 250 rpm. Cells from overnight cultures were harvested by centrifugation, resuspended in LB containing a final concentration of 25% (v/v) glycerol and frozen at −80 °C for storage.

### Transformation of *P. pastoris* expression strains

Competent *P. pastoris* (SuperMan_5_ or X33) cells were transformed essentially as previously described [40, 41] but using 100 ng DNA linearized by enzymatic digestion with *Bsp*HI (according to the NEB protocol) followed by DNA purification (NucleoSpin® Plasmid EasyPure Kit, Macherey–Nagel). After transformation by electroporation the cells were spread on YPD plates containing 10 µg/mL bleocin (Merck Millipore) and were incubated for 3 days at 30 °C. Eight colonies representing each construct were then transferred to a new plate containing the same medium.

### Expression screening in *E. coli*

Expression screening was carried out in 96-well plates (Eppendorf). Each well contained 300 µL LB medium plus 15 µg/mL gentamicin and was inoculated with overnight cultures of *E. coli* transformants to yield an OD_600_ of 0.1. The space between the wells was filled with water and the plate was sealed with Parafilm to minimize evaporation. The cells were cultivated in a plate reader (Biotek) at 37 °C for 3 h with linear shaking at 567 cpm and an amplitude of 3 mm. Absorption at 600 nm was measured online every 20 min to monitor cell growth. After 3 h, protein expression was induced by adding 1 mM IPTG or 13 mM arabinose, depending on the promoter. The cultivation temperature was lowered to 30 °C during the 17-h protein expression phase. Cells were harvested by centrifugation and lysed with BugBuster Master mix (Merck Millipore) following the manufacturer’s protocol. Lysates were stored at 4 °C. Proteins in the cell lysates were quantified by a His_6_ tag-specific sandwich ELISA using the His Tag Antibody Plate (GenScript) following the manufacturer’s protocol. Samples were diluted 1:100. Penta∙His HRP Conjugate (Qiagen) was diluted 1:4000 and was used as the detection antibody. The amount of target protein was calculated using a dilution series of His_6_ tag human ephrin-B1 (Sino Biological) for calibration.

### Expression screening in *P. pastoris*

Constitutive and methanol induction screening were carried out as previously described [42] but instead of humidity control, a double layer of Parafilm was added between the lid and the 96-deep-well plate. The starting volume was 500 µL of BMY medium containing 1% glucose. For the feed, the cells were pipetted up and down thoroughly to ensure good mixing and 125 µL medium was then removed and replaced with 125 µL fresh BMY medium containing 4% glucose every 24 h, making four replacements in total. Twenty-four hours after the last feed, the supernatant was harvested by centrifugation (10,000×*g*, 5 min) and 200 µL supernatant (undiluted) was used for protein quantification by a His_6_ tag-specific sandwich ELISA as described for *E. coli*.

## Additional file

**Additional file 1.** Soluble and insoluble cell lysate fraction after expression of Trx-BR021 in *E. coli* BL21 (DE3) in shaking flasks (SDS-PAGE).

## Abbreviations

AMP: antimicrobial peptide
GSL: gibberillin stimulated like peptide
IMPI: insect metalloproteinase inhibitor
TrxB: thioredoxin reductase B
gor: glutathione reductase
SLIC: sequence and ligation independent cloning
PCR: polymerase chain reaction
AFP: antifungal peptide
GST: glutathione-*S*-transferase
Trx: thioredoxin
Thr: thrombin
pGAP: glyceraldehyde 3-phosphate dehydrogenase promoter
pAOXI: alcohol oxidase I promoter
Kex2: the α-mating factor with an additional specific serine endoproteinase cleavage site
tAOXI: alcohol oxidase I terminator
