# Supplementary figures and images for: A high-throughput expression screening platform to optimize the production of antimicrobial peptides
Source: Microb Cell Fact. 2017 Feb 13;16:29. doi: 10.1186/s12934-017-0637-5 (PMC5307881; doi:10.1186/s12934-017-0637-5)

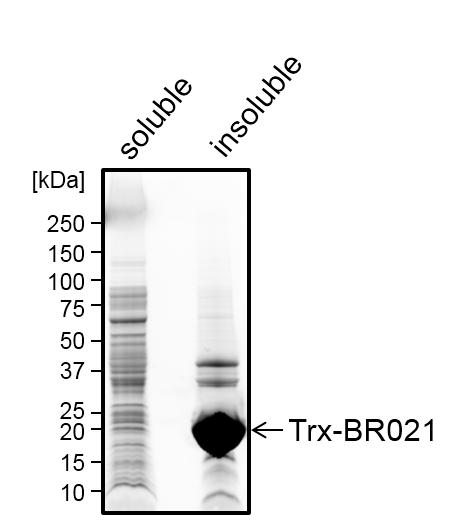

Supplement: Supplementary file 1 — Additional file 1. Soluble and insoluble cell lysate fraction after expression of Trx-BR021 in E. coli BL21 (DE3) in shaking flasks (SDS-PAGE). [file 12934_2017_637_MOESM1_ESM.tif]
